# 2-Butanol Aqueous Solutions: A Combined Molecular Dynamics and Small/Wide-Angle X-ray Scattering Study

**DOI:** 10.1021/acs.jpca.2c05708

**Published:** 2022-11-17

**Authors:** Marina Macchiagodena, Gavino Bassu, Irene Vettori, Emiliano Fratini, Piero Procacci, Marco Pagliai

**Affiliations:** † Dipartimento di Chimica “Ugo Schiff”, Università degli Studi di Firenze, Via della Lastruccia 3, 50019 Sesto Fiorentino, Firenze, Italy; ‡ Consorzio per lo Sviluppo dei Sistemi a Grande Interfase (CSGI), Via della Lastruccia 3, 50019 Sesto Fiorentino, Firenze, Italy

## Abstract

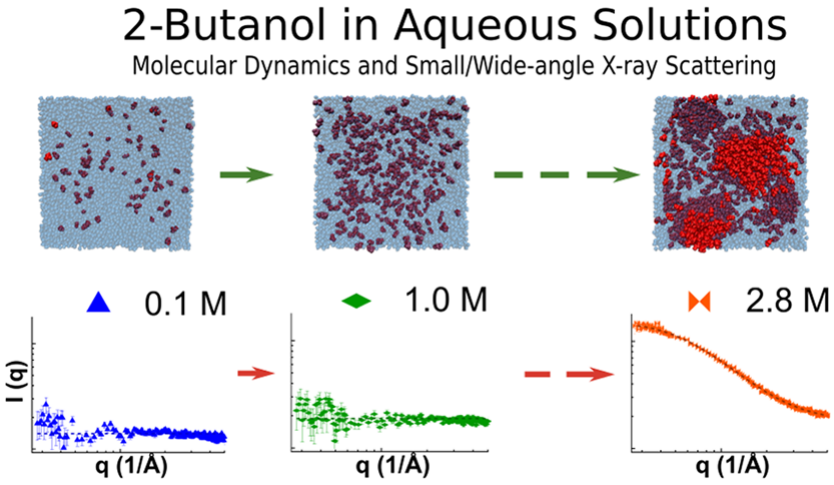

Structural properties of 2-butanol aqueous solutions at different concentrations have been studied using small- and wide-angle X-ray scattering and molecular dynamics simulations. The experimental structure factors have been accurately reproduced by the simulations, allowing one to explain their variation with concentration and to achieve a detailed description of the structural and dynamic properties of the studied systems. The analysis of experimental and computational data has shown that 2-butanol, the simplest aliphatic chiral alcohol, tends to form aggregates at a concentration above 1 M, affecting also both the structural and dynamic properties of the solvent.

## Introduction

Alcohols are amphiphilic molecules formed by two different moieties: one polar, characterized by the hydrophilic −OH group, and one apolar, corresponding to the aliphatic hydrocarbon chain. This characteristic affects both the structure of the alcohol in the aqueous solution and the dynamics of the surrounding water molecules. As the alcohol concentration increases, the system responds forming alcohol clusters.^1^

The alcohol aqueous solutions are widely studied for their specific properties and applications in numerous research, technological, and medical sectors.^2−5^

**Figure 1:**
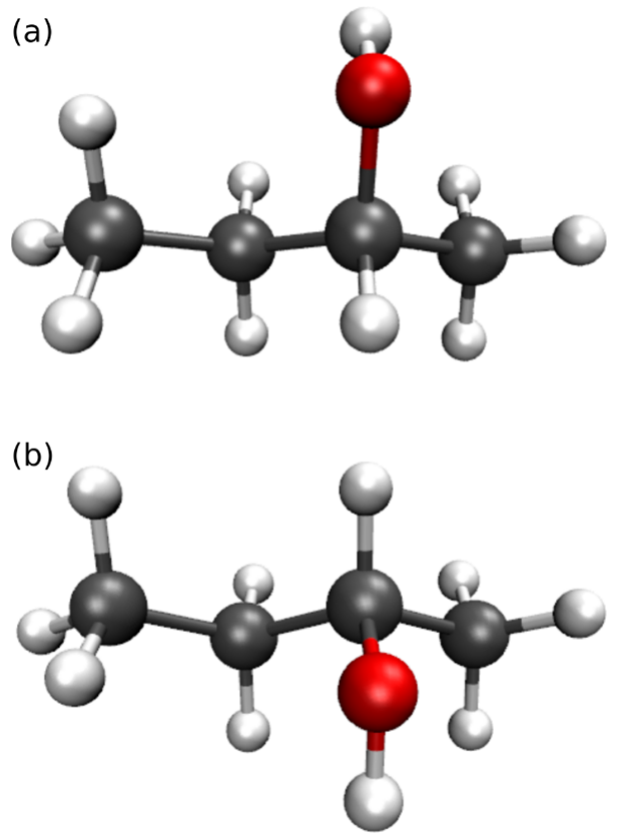
Molecular structure of (a) (*R*)-2-butanol and (b) (*S*)-2-butanol enantiomers.

In the present study, both experimental and computational results for 2-butanol aqueous solutions are reported. 2-Butanol is one of the isomers of butyl alcohol and the only chiral one. Two enantiomeric forms of 2-butanol ((*R*)- and (*S*)−) exist as shown in panels (a) and (b) of Figure 1, and in nature it is usually present as a racemic mixture.^2,6^

Among the butyl alcohol isomers, 2-butanol is the less studied one. In fact, interactions in 2-butanol-based systems have been only partially investigated by a computational approach, mainly through density functional theory (DFT) calculations, focusing on chirality aspects^7,8^ or on clathrate structure formation.^9^ The interest in studying 2-butanol relies mainly in its uses in the fuel industry, both as an additive^10,11^ and as a platform for biofuel production.^12,13^ Further fields of application regard the food industry,^14,15^ electrosorption,^16,17^ and hydrogenation routes.^18^

To characterize the structure and the dynamics of 2-butanol aqueous solutions, molecular dynamics (MD) simulations and Small/Wide-Angle X-ray Scattering (SWAXS) measurements have been carried out. The agreement between the experimental and the computational structure factors has been verified so as to validate the MD protocol and the chosen force field. Several studies were conducted to verify that the dimensions of the simulation box correctly consider the long-range intermolecular interactions and to assess the accuracy of force fields.^19−22^ In the present study, the semiquantitative reproduction of experimental findings allows us to adopt MD simulations with great confidence. This result is fundamental to provide an accurate description, at an atomic level, of the intermolecular interactions in 2-butanol solutions (considering a racemic composition of (*R*)- and (*S*)-enantiomers). Particular attention has been devoted to the known clustering tendency of butanol isomers at the mesoscale with increasing alcohol concentration.^23^ Therefore, the results are of interest in providing information on both 2-butanol cluster formation while disclosing its effect on the structural and dynamic properties of water.

## Methods

### Sample Preparation

The solutions were prepared by directly mixing 2-butanol (Merck; assay > 99%) and ultrapure Milli-Q water (18.2 M Ω·cm). The solutions were stored at 20 °C and strongly mixed before each use.

### Small- and Wide-Angle X-ray Scattering

Small- and Wide-Angle X-ray Scattering (SWAXS) analyses were carried out using a Xeuss 3.0 HR apparatus (Xenocs, France), equipped with a movable EIGER2R (1 M model) hybrid pixel photon counting detector (Dectris Ltd., Switzerland) consisting of 1028 × 1062 pixels with a size of 75 × 75 μm^2^. The X-ray beam corresponds to the *Kα* radiation (λ = 1.5406 Å) emitted by a Cu microfocus (30W) sealed tube operated by a Genix 3D generator. The average acquisition time per sample was 600 to 1200 s. The calibration of the sample to detector distance was performed using the scattering pattern of a well-known lamellar phase (i.e., silver behenate, *d* = 58.376 Å).^24^ The pure solvents and 2-butanol aqueous solutions were analyzed in sealed borosilicate glass capillaries of 1 mm of internal diameter and at 20 °C. The experiments were performed under vacuum to minimize the scattering from air, using three sample-to-detector distances (i.e., 80, 400, and 1800 mm) to cover a continuous *q*-range interval going from 0.004 to 2.775 Å^–1^. The scattering vector, *q*, is defined as *q* = 4π/λ sin θ, where 2θ represents the scattering angle. Data reduction, normalization, subtraction, and merging were performed using XSACT (X-ray Scattering Analysis and Calculation Tool) software (Xenocs, France). In particular, each curve was radially averaged and corrected for the scattering of the empty capillary according to the relative transmission factors. The scattering curves were, then, converted in absolute intensity (mm^–1^), by measuring the scattering intensity distribution of a glassy carbon reference specimen in the same experimental conditions.^25^ SWAXS was performed on 2-butanol solutions with concentrations of 0.1, 0.5, 1.0, 2.0, 2.5, and 2.8 M and on pure water and 2-butanol.

### Density Measurements

The density of each solution was measured using a Mettler Toledo DA-100 M Density Meter at a constant temperature of 20.0 ± 0.1 °C. The instrument allows the determination of the density from 0 to 3000 kg/m^3^ with an accuracy of 1 kg/m^3^.

### Computational Details

MD simulations were performed using GROMACS 2021.2 software^26^ on the Marconi 100/CINECA HPC system.^27^ A racemic solution of 2-butanol was solvated in a cubic box with TIP4P-FB^28^ water molecules. The number of solute/solvent molecules was chosen to obtain concentrations of 0.1, 0.5, 1.0, 1.5, 2.0, 2.5, and 2.8 M comparable with experimental measurements (details are summarized in Table 1). In addition, a simulation of 2048 TIP4P-FB water molecules was performed to compare water dynamic properties.

**Table 1:** Molecular Dynamics Simulation Details

	number of molecules		
concn (M)	solute^a^	solvent	average box length (Å)
0.1	80	43986	110.0
0.5	400	42488	110.0
1.0	802	40617	110.1
1.5	1202	38750	110.2
2.0	1604	36830	110.3
2.5	2004	35003	110.5
2.8	2244	33880	110.6

a Composed of an equal number of (*S*)- and (*R*)-enantiomers.

The Generalized Amber Force Field (GAFF2) for (*R*)- and (*S*)-2-butanol was assigned using the PrimaDORAC web interface.^29^ The atomic charges were parametrized by using both the standard AM1-BCC charges obtained with PrimaDORAC^29^ and those obtained by a CM5 population analysis^30^ at the B3LYP/6-31+G(d) level of theory, considering the mean solvent polarization effects through C-PCM.^31^ This second protocol has been successfully adopted in modeling both pure solvents and molecules in solution.^32−34^ The comparison between AM1-BCC and CM5 atomic charges is reported in Table S1 of the Supporting Information (SI) to highlight the differences in the electrostatic description behind these approaches. Since GAFF2 provides results in better agreement with experiments (see for example Table 2), this force field has been adopted in the manuscript as a reference to support the experimental findings.

**Table 2:** Calculated Densities from 10 ns NPT Simulation and Experimental Values^a^

	density (kg/m^3^)		
concn (M)	computed	experimental	error (%)
0.1	995.65 ± 0.02	996 ± 1	0.04
0.5	991.30 ± 0.02	992 ± 1	0.07
1.0	985.59 ± 0.03	987 ± 1	0.1
1.5	976.32 ± 0.24	982 ± 1	0.6
2.0	968.06 ± 0.67	976 ± 1	0.8
2.5	957.95 ± 0.85	970 ± 1	1.3
2.8	952.16 ± 0.88^b^	967 ± 1	1.6

a The experimental densities were measured for 0.1, 0.5, 1.0, 2.0, and 2.8 M and linearly interpolated for the intermediate concentrations (i.e., 1.5 and 2.5 M).

b Using the CM5 atomic charges, we obtained a value of 949.26 ± 0.09 (kg/m^3^).

The systems were initially minimized at 0 K with a steepest descent procedure and subsequently thermalized at 298.15 K keeping the temperature constant with a Nosé–Hoover thermostat,^35,36^ while the external pressure was set to 1 atm using the Parrinello–Rahman^37^ method. The time length of the equilibration run was 1 ns with an integration time step of 0.1 fs. Production runs in the NPT ensemble were carried out for 10 ns imposing rigid constraints only on the X-H bonds (with X being any heavy atom) by means of the LINCS algorithm (δ*t* = 2.0 fs).^38^ Electrostatic interactions were treated via the particle-mesh Ewald (PME)^39^ method with a grid spacing of 1.2 Å and a B-spline interpolation of the 4*th* order. As prescribed by the AMBER protocol, the cross interactions for Lennard-Jones terms were calculated using the Lorentz–Berthelot^40,41^ mixing rules, and we excluded intramolecular nonbonded interactions between atom pairs separated up to two bonds. The nonbonded interactions between 1 and 4 atoms involved in a proper torsion were scaled by the standard AMBER fudge factors (0.8333 and 0.5 for the Coulomb and Lennard-Jones, respectively).

MD simulations were analyzed using both GROMACS tools^26^ and TRAVIS software,^42,43^ which was also used to derive the computed structure factor^44^ according to eq 1
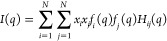
1where *H*_*ij*_(*q*), the partial structure factors, are calculated using eq 2
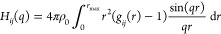
2

In eqs 1 and 2, *r* and *q* are the distance and the wave vector modulus, respectively. The indices *i* and *j* run over the N different atom types in the simulations; *x*_*i*_ is the mole fraction of atom type *i*, *f*_*i*_(*q*) is the atomic scattering factor of atom type *i*, and *g*_*ij*_(*r*) is the radial distribution function of atom types *i* and *j*. The number density of atoms in the simulation is denoted by ρ_0_, and *r*_max_ is the maximum sampled distance in the radial distribution function.

The normalized molecular concentration, *R*, was derived by the average number of 2-butanol molecules surrounding a reference one, considering a sphere of an 18 Å radius and the number of 2-butanol molecules found in the uniform distribution as the normalization factor, according to eq 3:
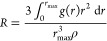
3In eq 3, *r*_max_ has been imposed equal to 18 Å, ρ is the 2-butanol density, and g(r) is the radial distribution function calculated between the center of mass (COM) of 2-butanol molecules. This quantity was used to disclose the increase in butanol/butanol interaction with concentration (i.e., clustering tendency).

## Results and Discussion

MD simulations allow the description of the physicochemical properties of a variety of complex systems. However, the choice of the force field is not always straightforward, and validation steps against real experiments are often required. Table 2 lists the MD calculated densities of the water/2-butanol solutions as compared to the experimental ones. The reported values show the agreement between experimental and computational results, with an increasing error that remains within 2% even in the case of concentrations close to the phase separation.^23^

**Figure 2:**
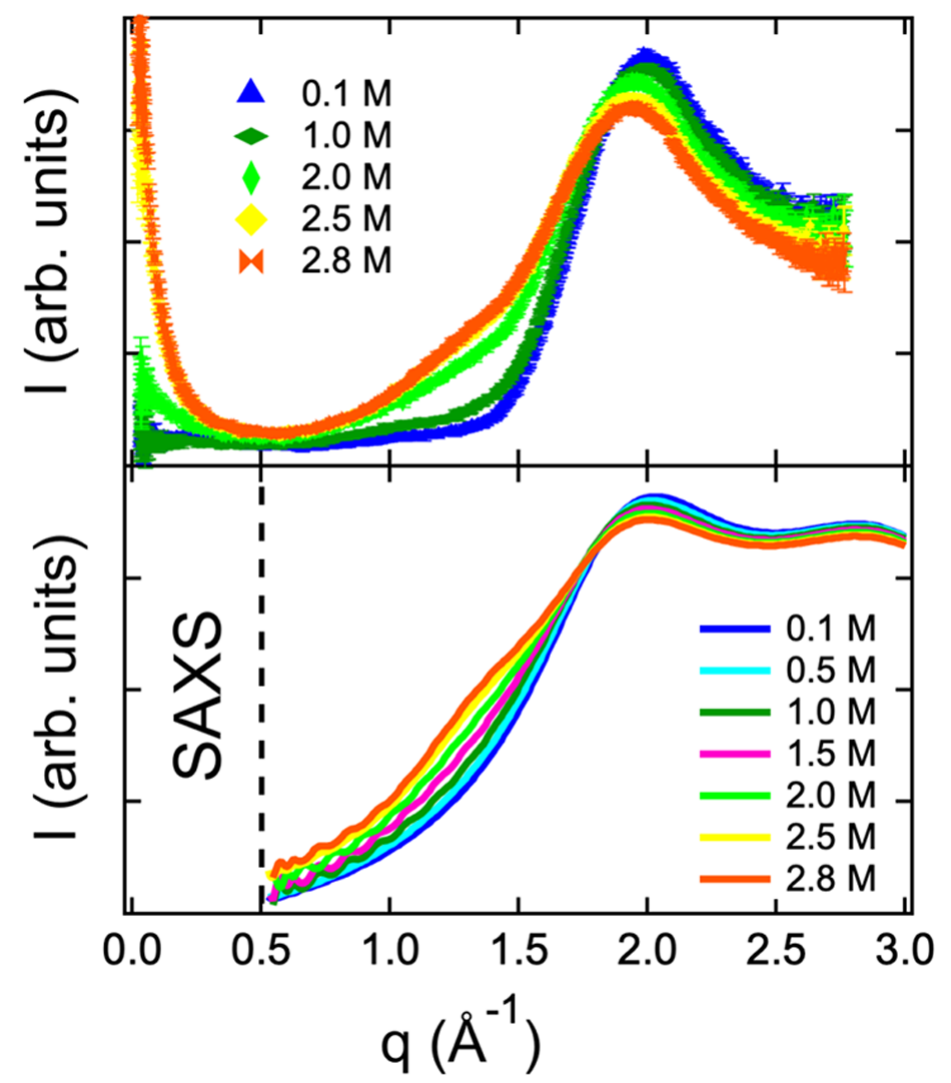
Experimental (up) scattering profile and computed structure factor (down) for 0.1 (blue), 0.5 (cyan), 1.0 (dark green), 1.5 (magenta), 2.0 (green), 2.5 (yellow), and 2.8 (orange) M 2-butanol solutions.

The spatial microscopic arrangement in 2-butanol aqueous solutions has been investigated by SWAXS and compared with the computed MD structure factor. The result is reported in Figure 2 (and in log–log scale in Figure S2 of the SI). In addition, Figure S2 of the SI shows the SWAXS patterns for pure water (violet line) and 2-butanol (red line). The pure 2-butanol SWAXS pattern exhibits two distinct peaks, the main diffraction peak and a secondary peak usually referred to as prepeak feature.^45^ The latter can be observed at about 0.7 Å^–1^, while the main diffraction maximum is at about 1.4 Å^–1^. In the case of the mono-ols series, Pozǎr et al.^45^ found a systematic decrease of the prepeak position with increasing alcohol length. In the case of the 2-butanol aqueous solutions, this prepeak is lost, and the diffraction patterns are dominated by the main structural peaks of water and 2-butanol. The profiles are in agreement with the expected trend obtained increasing 2-butanol concentration.

**Figure 3:**
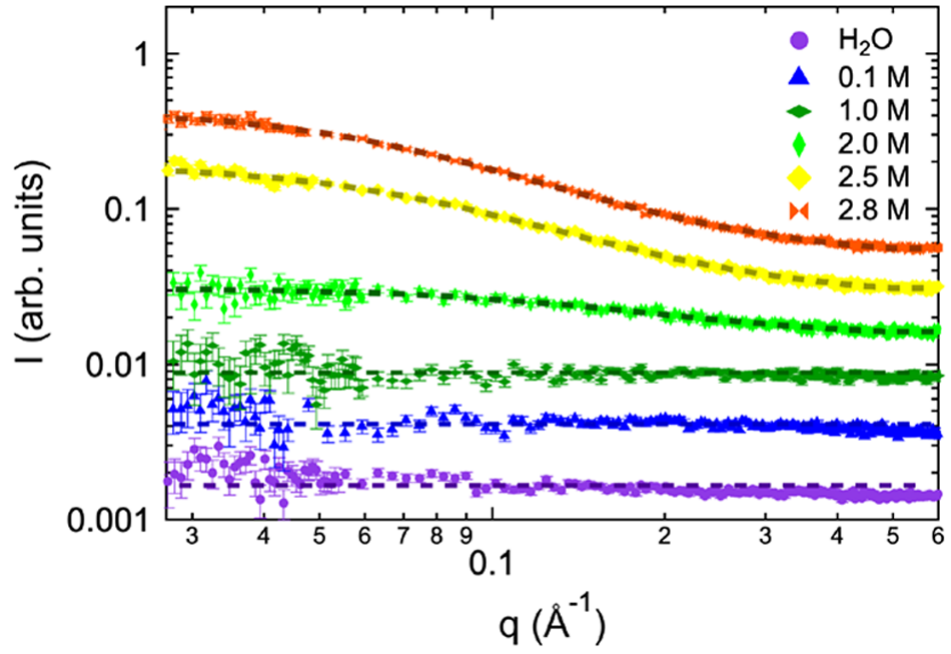
Fitting SAXS data at low *q* values for 0.1 (blue), 1.0 (dark green), 2.0 (green), 2.5 (yellow), and 2.8 (orange) M 2-butanol solutions as well as neat water (violet). SAXS curves have been shifted along the *y*-axis for the sake of clarity.

As shown in Figure 2, the calculations accurately reproduce the experimental finding in the 0.5–3 Å^–1^ range. Although the normalization factors are different, a semiquantitative agreement is evident. It is interesting to note that both the experimental and computed profiles, in the wide angle region, show evidence for alcohol aggregation when the concentration is greater than 1 M. In fact, a shoulder in both the experimental and computational profiles appears around 1.25*–*1.50 Å^–1^ due to the heterogeneity of the solution which increases with 2-butanol concentration. These findings have been verified by factorizing the computed structure factor for the 2.8 M solution into partial contributions. The intramolecular contributions have been initially separated from the intermolecular ones, while with a subsequent analysis, the intra- and intermolecular contributions related to each species have been computed. The results are shown in Figure S3 of the SI. In particular, panel (c) of Figure 3 reveals that the shoulder is due to the 2-butanol intermolecular interactions, confirming the presence of alcohol aggregation at higher concentrations. A similar profile has been found for *t*-butanol/water solutions (0 ≤ χ_*t*-*butanol*_ ≤ 1). For these systems, a peak occurs at 1.307 Å^–1^, which gradually shifts to 1.251 Å^–1^ by increasing the water content, and it has been attributed to interactions between *t*-butanol alkyl tails.^19^

SWAXS patterns in the small angle region (i.e., <0.50 Å^–1^) either in the lin-lin (see upper panel of Figure 2) and more clearly in the log–log (see Figure 3) representation unambiguously confirm the presence of clusters for 2-butanol concentration values of about 2 M or higher. In binary mixtures of small molecules, as those investigated in the present case, the scattering function at small *q* is described by the well-known Ornstein–Zernike (OZ) function^46^
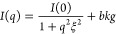
4where *I*(0) is the forward scattering intensity, ξ is the average correlation length proper of the aggregate, and *bkg* is the *q*-independent part of the scattering at small *q*, which arises due to the incoherent scattering always present for liquids. If we consider an aggregate with a spherical shape, its average radius can be calculated^47^ from the extracted correlation length as , while the corresponding radius of gyration results are . The contribution of the aggregate to the forward scattering is negligible for 2-butanol concentrations up to 1 M, while it increases for higher concentrations. In particular, the average aggregate radius passes from about 1 to about 3 nm as the concentration increases toward the phase separation. To verify whether the adopted model in MD simulations is able to reproduce the OZ behavior, further calculations on the 2.0 M sample concentration have been carried out increasing twice the simulation box along each direction (box length of 220 Å, 12832 solute molecules, 294640 solvent molecules). This new MD simulation allows the evaluation of the scattering function at small *q* as shown in Figure S4 of the SI. Although the box length is not sufficient to cover all of the *q* range of the experiment, the trend is in agreement with measurements. In the case of the pure water profile, the forward scattering values in absolute intensity provide a scattering cross section factor of 1.69 × 10^–3^ mm^–1^ which allows one to obtain the isothermal compressibility coefficient (eq 4 of ref (48)) in good agreement with the literature.^49^

**Figure 4:**
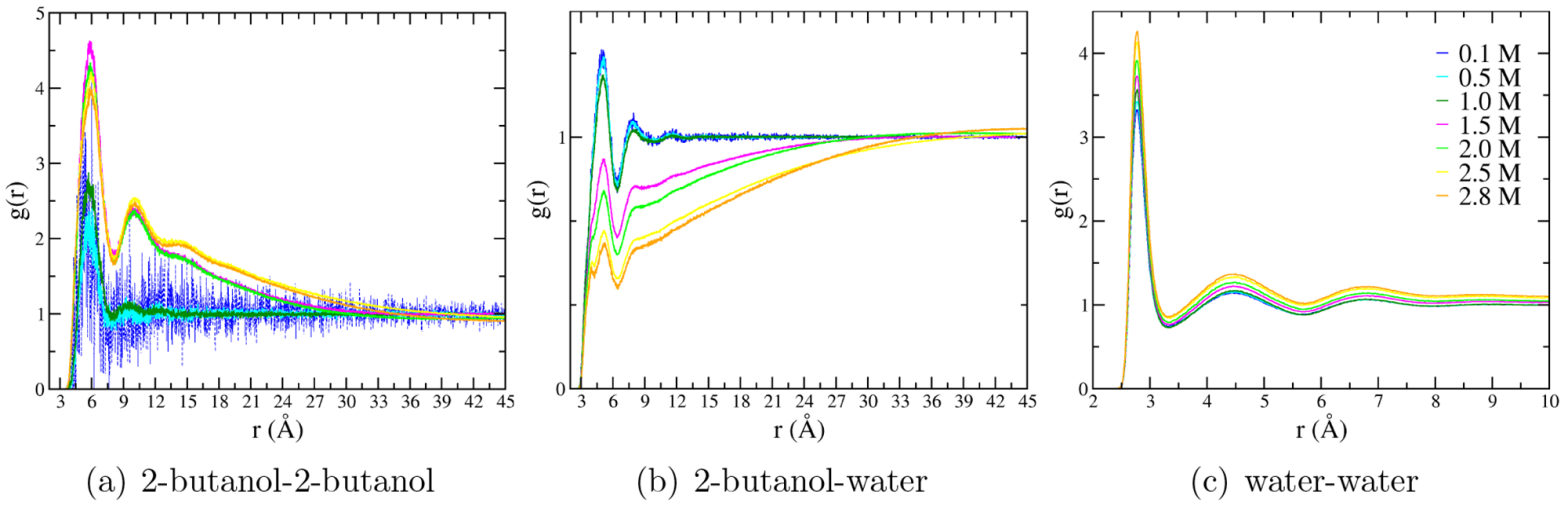
Radial distribution function between COMs for (a) 2-butanol-2-butanol, (b) 2-butanol-water, and (c) water–water interactions, respectively. The radial distribution functions have been reported for the different 2-butanol concentrations: 0.1 (blue), 0.5 (cyan), 1.0 (dark green), 1.5 (magenta), 2.0 (green), 2.5 (yellow), and 2.8 (orange) M 2-butanol solutions.

Since the MD allows the reproduction of experimental structural data, MD trajectories have been employed to disclose the effect of the concentration on both structural and dynamic properties in the 2-butanol aqueous solution. The interactions involving 2-butanol have been obtained by computing the pair radial distribution functions, g(r), related to the COM of the solute and solvent molecules (Figure 4).

The g(r) related to the 2-butanol/2-butanol interactions with the concentration shows in Figure 4(a) a different trend above 1 M. In the case of a concentration of about 1.5 M or higher, the interactions between alcohols became dominant, allowing one to hypothesize the formation of 2-butanol clusters. This hypothesis is corroborated by analyzing the solute/solvent interactions in Figure 4(b). The height of the first peak in g(r), related to the COM of both water and 2-butanol, decreases with the concentration. Also in this case, the g(r) trend is similar for the 2-butanol concentration lower than 1.5 M, whereas starting from this value, it is possible to note a rapid decrease of the first peak height. Moreover, at concentrations higher than 1.5 M, the first peak splits into two contributions at about 4 and 5 Å (as it can be appreciated by the magnification of Figure 4(b) in Figure S5 of the SI), which can be due to different 2-butanol/water interactions affecting both structure and dynamics properties. Increasing the concentration of 2-butanol, the interactions between solute–solvent molecules decrease, whereas those between molecules of the same specie increase. Figure 4(c) shows that g(r) related to water–water interactions is more structured at higher than at lower concentration. This behavior can be considered as a result of the hydrophobic interaction with the alcohol: thus, the water molecules are compressed and obliged to stay closer to each other.

**Figure 5:**
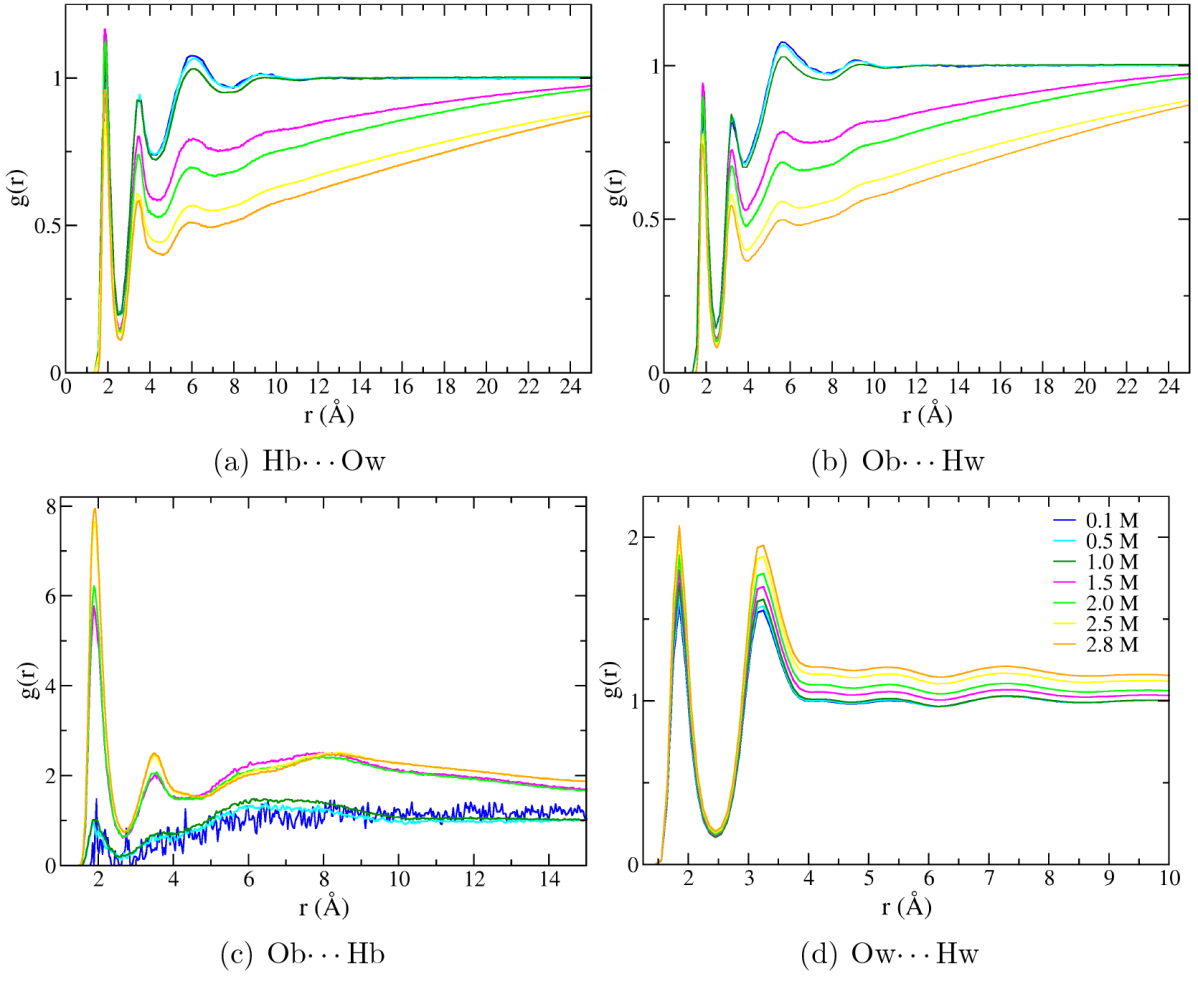
Radial distribution function related to hydrogen bond interactions: (a) H atom of the alcoholic group of 2-butanol (Hb) with the oxygen atom of water (Ow), (b) O atom of the alcoholic group of 2-butanol (Ob) with the hydrogen atom of water (Hw), (c) 2-butanol-2-butanol interactions (Ob···Hb), and (d) water–water interactions (Ow···Hw), respectively. The radial distribution functions have been reported for the different 2-butanol concentrations: 0.1 (blue), 0.5 (cyan), 1.0 (dark green), 1.5 (magenta), 2.0 (green), 2.5 (yellow), and 2.8 (orange) M 2-butanol solutions.

In this respect, further information on the intermolecular interactions can be obtained by the analysis of the g(r) related to the hydrogen bonds involving solute–solute, solute–solvent, and solvent–solvent, as shown in Figure 5. As expected, considering both the acceptor and donor hydrogen bond interactions of 2-butanol with water, the probability to find solute–solvent interactions decreases with the concentration of the alcohol. Moreover, it is possible to appreciate in panels (a) and (b) of Figure 5, that starting from 1.5 M, the curves are less prone to reach the value of 1, i.e., the uniform distribution of the solvent. On the contrary, the 2-butanol-2-butanol aggregation can be appreciated in panel (c) of Figure 5 as confirmed also in this case by the different trends for a concentration higher than 1.0 M. Finally, panel (d) of Figure 5 shows that there is a higher probability of finding oxygen–hydrogen interactions for water molecules, as a consequence of the increasing structure of the solvent imposed by the hydrophobic effect.

**Figure 6:**
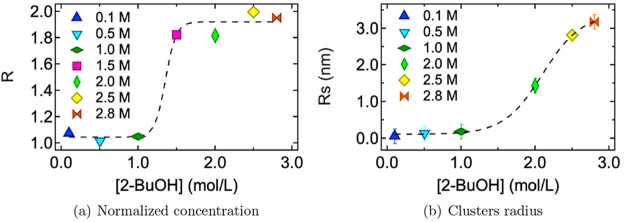
(a) *R* (see text) as a function of 2-butanol concentration: 0.1 (blue), 0.5 (cyan), 1.0 (dark green), 1.5 (magenta), 2.0 (green), 2.5 (yellow), and 2.8 (orange) M. (b) Cluster dimension obtained fitting the SAXS patterns with eq 4 which correspond to the OZ description of simple liquids. *Rs* has been determined for the different 2-butanol aqueous solutions: 0.1 (blue), 0.5 (cyan), 1.0 (dark green), 2.0 (green), 2.5 (yellow), and 2.8 (orange) M. Dashed lines in both panels represent the best fit according to a sigmoid function (eqs 1 and 2 of the SI) where the inflection, representing the transition point, results are 1.0 M (a) and 2.1 M (b), respectively.

The behavior observed in the g(r) analysis can be further supported by considering the normalized molecular concentration, *R*, at the different concentration values. This approach allows one to determine at which concentration a significant deviation from the uniform distribution of 2-butanol molecules is observed. As can be appreciated from Figure 6(a) when the 2-butanol concentration becomes greater than 1 M, *R* approaches the value of 2, meaning that the average number of 2-butanol molecules in a sphere of 18 Å is two times higher than in a uniform distribution; these deviations are due to the aggregation of 2-butanol molecules forming clusters. To estimate the correlation length of the clusters, ξ, the OZ model^46^ has been applied on SAXS curves. The results are reported in Figure 6(b) considering the geometrical radius of a spherical aggregate, *Rs*. Although Figure 6(a) and Figure 6(b) display different information, in one case the 2-butanol distribution and the second one the clusters dimension, a similar trend is evident: when the 2-butanol concentration is higher than 1 M, the alcohol molecules start to form aggregates. Furthermore, the comparison in Figure 6 allows one to confirm the good agreement between the experimental and the computational approach, showing that a synergistic combination of them is possible that allows for a detailed interpretation of the structure and dynamics of the system.

**Figure 7:**
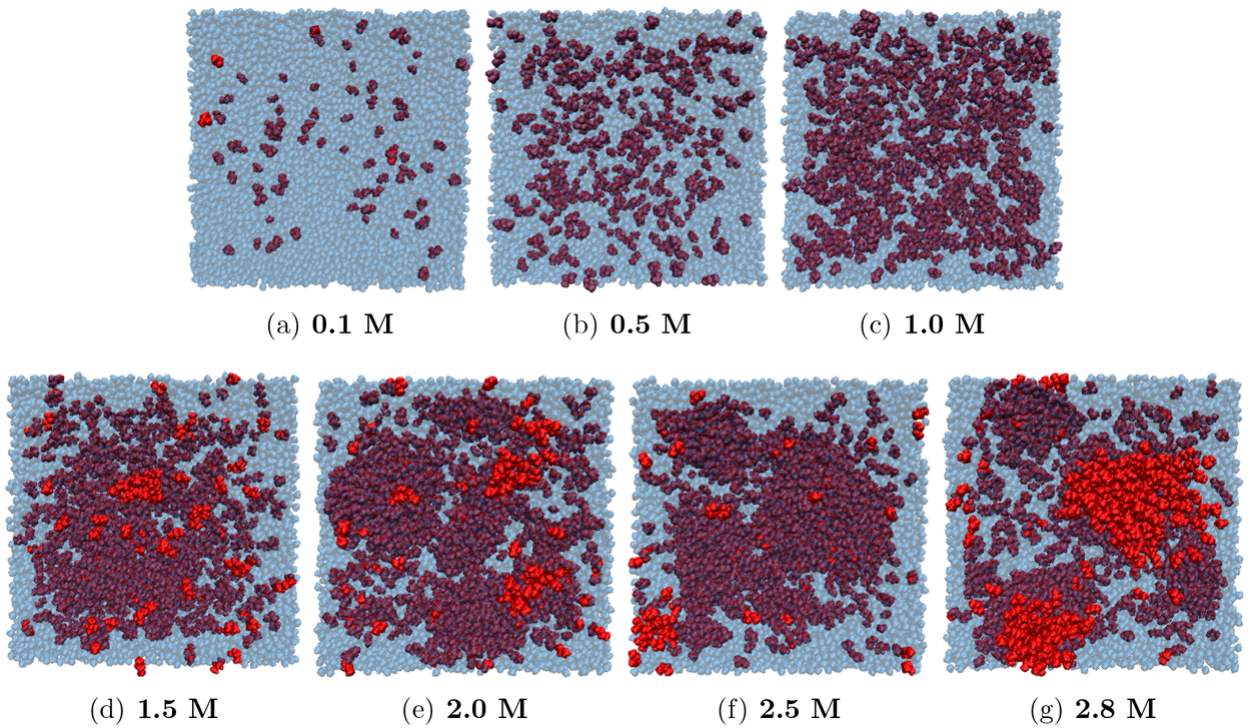
Snapshot of simulated systems: in red solute molecules, in blue transparent water molecules.^50^ The concentration of 2-butanol is indicated by the number below each panel. The edge of the simulation box is about 11 nm.

A graphical representation of possible clusters is reported in Figure 7; the snapshots of simulated systems allow the visualization of the cluster formation as the 2-butanol concentration is increased.

**Figure 8:**
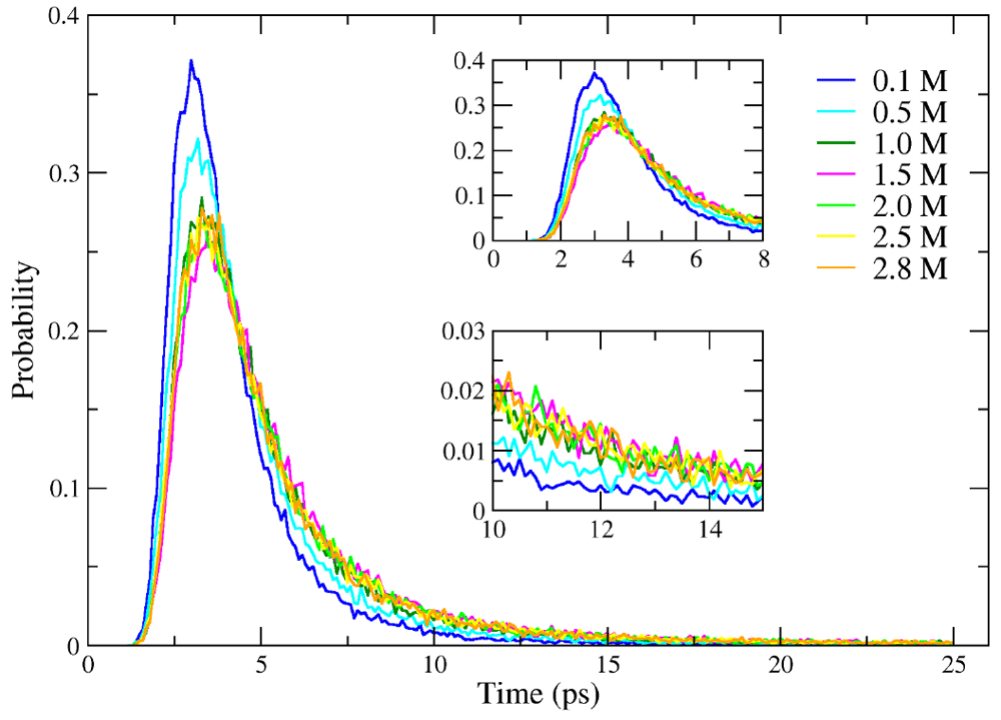
Distribution of time with an ACF of the water dipole moment equal to 0.5. The distribution has been calculated for different 2-butanol concentrations: 0.1 (blue), 0.5 (cyan), 1.0 (dark green), 1.5 (magenta), 2.0 (green), 2.5 (yellow), and 2.8 (orange) M. Upper and lower insets show the magnification of the short and long time regions, respectively.

As already reported, in Figure 4(b) (and in Figure S5 of the SI), the first peak of g(r) shows a bimodal distribution as the concentration approaches 1.5 M; this result could be attributed to different hydrogen bond interactions of the water molecules. The hypothesis has been verified computing the autocorrelation function (ACF) of the water dipole moment and plotting its distribution as a function of time only when the ACF reaches the value of 0.5. Figure 8 shows the effect of 2-butanol aggregation on the water molecule dynamics. The water molecules slow down with an increasing solute concentration due to the interaction with the solute imposed by the hydrophobic effect. For comparison in Figure S6 of the SI, we reported the same distribution in the case of pure water using the TIP4P-FB water model. As expected, the distribution is more narrow and peaked at a time value of 3.6 ps which is comparable with the relaxation time already reported.^51^

## Conclusions

SWAXS measurements and MD simulations have been performed on 2-butanol aqueous solutions. MD simulations accurately reproduce the experimental finding in the wide angle region and allow further characterization of the structural properties of selected 2-butanol aqueous solutions. With an increase in the 2-butanol concentration, it has been observed that the alcohol tends to form aggregates as the concentration is greater than 1.0 M. The experimental findings in the small angle scattering region allow the determination of the average cluster dimension (ξ) and the associated radius in the hypothesis of a spherical aggregate, *Rs*, at a concentration higher than 2 M. *Rs* shows a sigmoidal increase up to 3 nm for concentrations close to the phase separation with an inflection point at 2.1 M. This trend discloses a cooperative formation of the cluster as a result of the balance among the hydrogen bonding and the hydrophobic interactions. Moreover, it matches with cluster dimensions of about 6 nm evident in the simulation box reported in Figure 7. The presence of an aggregate in aqueous solution affects the dynamics of water, determining a slowdown of the orientational dynamics of the water dipole moment. The modifications are highlighted analyzing both experimental and computational derived data. MD simulations support the experimental finding of SWAXS measurements, showing how the synergy between experiments and computations can be adopted to reveal important structural and dynamics details.

## Supporting Information Available

AM1-BCC and CM5 atomic charges calculated for 2-butanol, log–log plot of experimental scattering profile, computed structure factor for 2.0 M 2-butanol solution, sigmoidal function used in fittings, magnification of Figure 4(b), and distribution of times with ACF of water dipole moment equal to 0.5 calculated using simulation of 2048 TIP4P-FB water molecule (PDF)
