# Supplementary material for: 2-Butanol Aqueous Solutions: A Combined Molecular Dynamics and Small/Wide-Angle X-ray Scattering Study
Source: J Phys Chem A. 2022 Nov 17;126(47):8826–33. doi: 10.1021/acs.jpca.2c05708 (PMC9720721; doi:10.1021/acs.jpca.2c05708)
Supplement: Supplementary file 1 — jp2c05708_si_001.pdf [file jp2c05708_si_001.pdf]

Supporting Information for "2-Butanol in Aqueous Solutions: a Combined Molecular Dynamics and Small/Wide-Angle X-ray Scattering Study"

Marina Macchiagodena,[†] Gavino Bassu,[†,‡] Irene Vettori,[†,‡] Emiliano Fratini,[*,†,‡] Piero Procacci,[†] and Marco Pagliai[*,†]

†*Dipartimento di Chimica "Ugo Schiff", Università degli Studi di Firenze, Via della Lastruccia 3, 50019 Sesto Fiorentino (FI), Italy*

‡*Consorzio per lo Sviluppo dei Sistemi a Grande Interfase (CSGI), Via della Lastruccia 3, 50019 Sesto Fiorentino (FI), Italy*

E-mail: emiliano.fratini@unifi.it; marco.pagliai@unifi.it

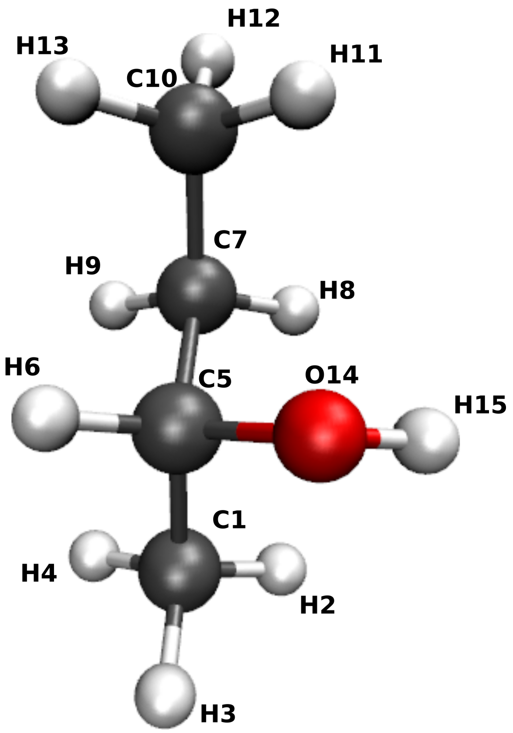

Figure S1: Atoms labeling used in table S1.

Table S1: AM1-BCC and CM5 atomic charges (e), see Figure S1 for the atoms correspondence in the molecular formula.

Atom	AM1-BCC	CM5
C1	-0.1288	-0.2315
H2	0.0420	0.0831
H3	0.0420	0.0831
H4	0.0420	0.0831
C5	0.1347	0.0075
H6	0.0750	0.0989
C7	-0.1121	-0.1555
H8	0.0423	0.0872
H9	0.0423	0.0872
C10	-0.0912	-0.2326
H11	0.0377	0.0757
H12	0.0377	0.0757
H13	0.0377	0.0757
O14	-0.5962	-0.4734
H15	0.3947	0.3358

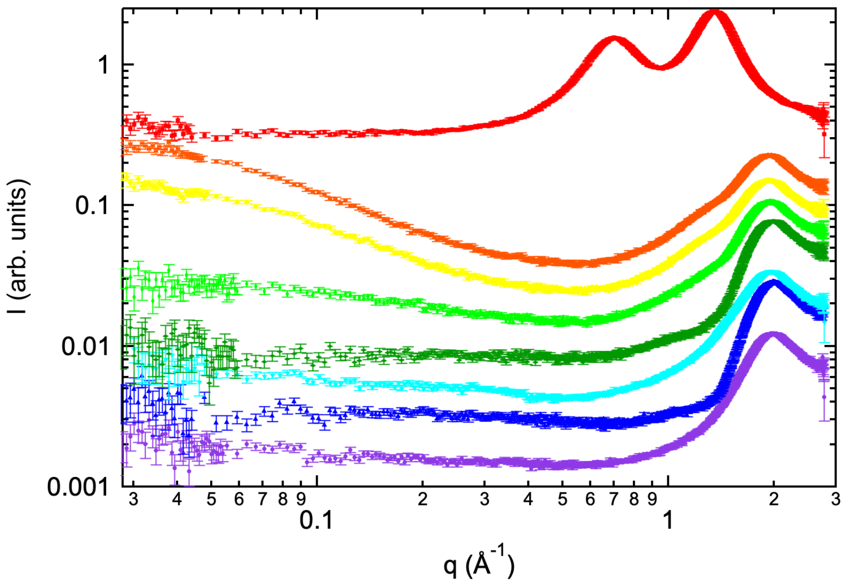

Figure S2: Log-log plot of experimental scattering profiles for the complete set of sample investigated: 0.1 (blue), 2.0 (green), 2.5 (yellow) and 2.8 (orange) M 2-butanol solutions as well as neat 2-butanol (violet) and water (red). SWAXS curves have been shifted along the y-axis for the sake of clarity.

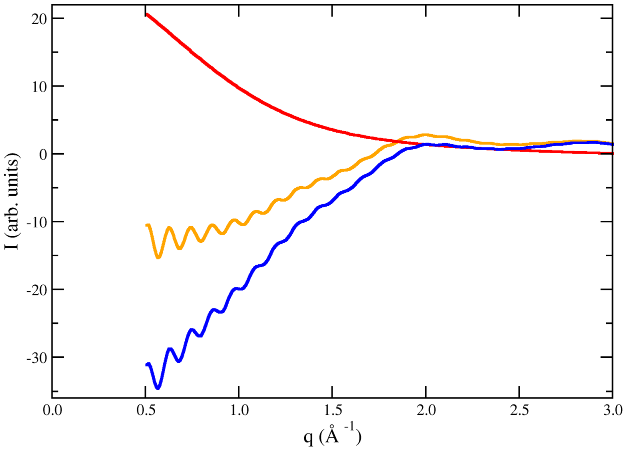

(a) Inter and intramolecular contributions.

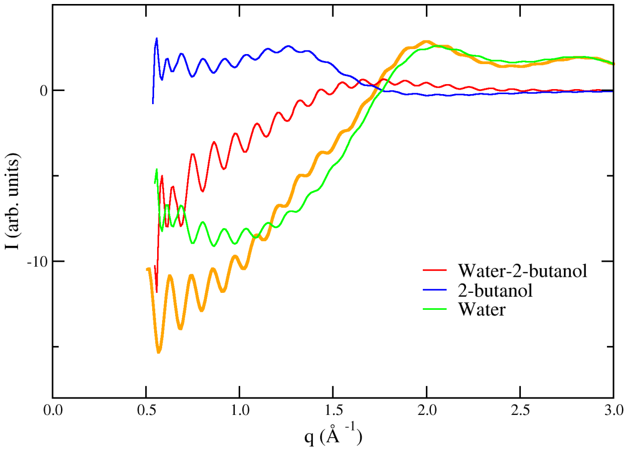

(b) Water, 2-butanol and water-2-butanol contributions.

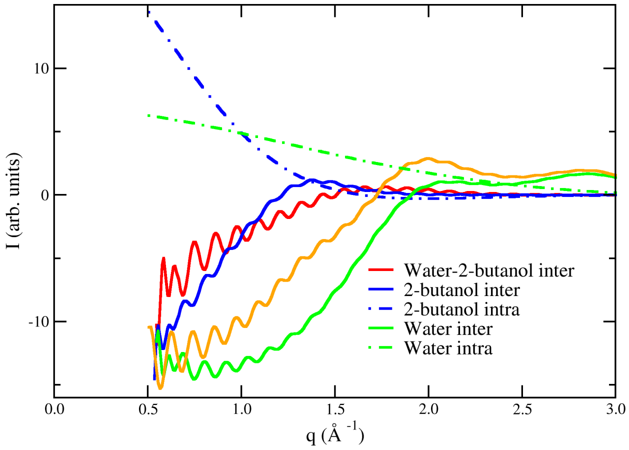

(c) Inter and intramolecular contributions.

Figure S3: (a) In orange computed structure factor, in blue the intermolecular contribution, in red the intramolecular contribution; (b) in orange computed structure factor, in red the contribution due to water-2-butanol interactions, in blue the contribution due to 2-butanol interactions and in green the contribution due to water interactions; (c) in orange computed structure factor, in red the contribution due to water-2-butanol interactions, in blue solid line the contribution due to 2-butanol intermolecular interactions, in blue dashed line the contribution due to 2-butanol intramolecular interactions, in green solid line the contribution due to water intermolecular interactions and in green dashed line the contribution due to 2-butanol intramolecular interactions. All data refer to 2-butanol 2.8 M solution.

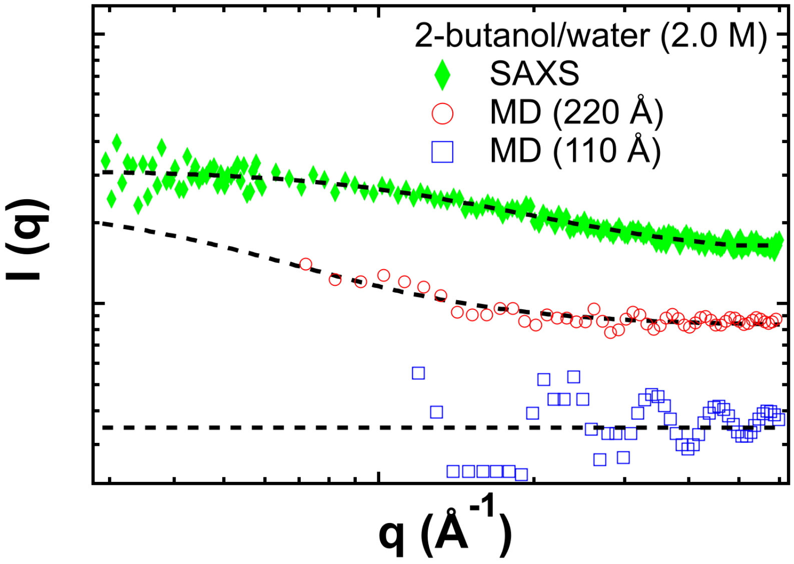

Figure S4: Experimental (green) and computed (red and blue) structure factor for 2.0 M 2-butanol solution. The computed $I(q)$ has been derived from two different simulation boxes: box length of 110 Å (blue squares) and 220 Å (red circles). Dashed lines represent the best fits according to the OZ model. Rs values extracted by the OZ model (see Equation 4 of the manuscript) are 1.4±0.2 nm and 3.1±2.1 nm in the experimental and computed case, respectively. The box length of 220 Å is still limited to achieve a precise estimation of the Rs value in the case of 2.0 M 2-butanol solution as testified by the extracted standard deviation.

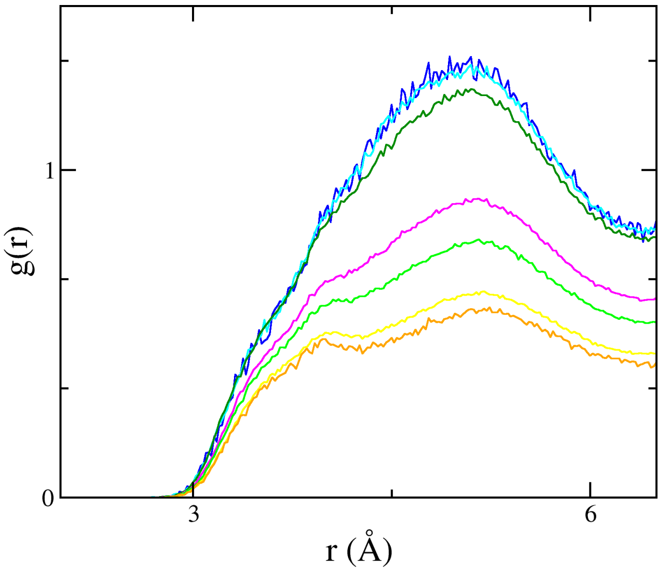

Figure S5: Magnification of Figure 4(b).

Sigmoidal function used to fit data in Figure 6:

$$Rs = base + \frac{max}{1 + e^{\frac{xhalf - [2-BuOH]}{rate}}} \tag{1}$$

with

$base = 0.102 \pm 0.028$

$max = 3.292 \pm 0.124$

$xhalf = 2.100 \pm 0.026$

$rate = 0.262 \pm 0.029$

$$R = base + \frac{max}{1 + e^{\frac{xhalf - [2-BuOH]}{rate}}} \tag{2}$$

with

$base = 1.046 \pm 0.056$

$max = 0.874 \pm 0.073$

$xhalf = 1.355 \pm 0.376$

$rate = 0.070 \pm 0.180$

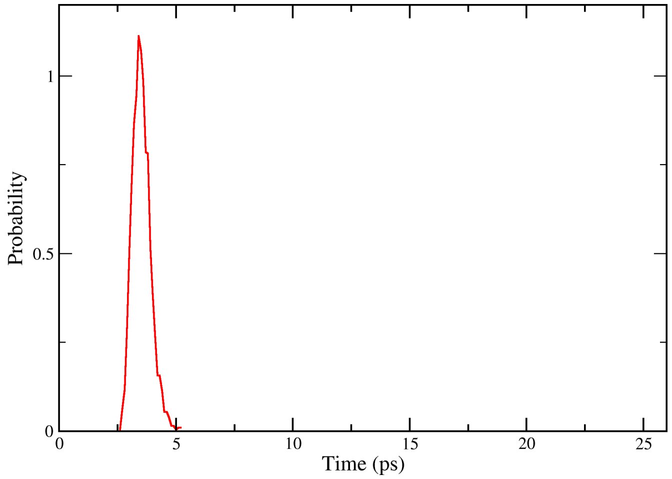

Figure S6: Distribution of times with a ACF of water dipole moment equal to 0.5 for pure water. The distribution has been calculated using a simulation of 2048 TIP4P-FB water molecule.
